# Bis[μ-2-(2,4-difluoro­phen­yl)-1,3-bis­(1,2,4-triazol-1-yl)propan-2-olato-κ^4^ *N* ^2^,*O*:*O*,*N* ^2′^]bis­[(acetato-κ^2^ *O*,*O*′)nickel(II)] methanol hemisolvate

**DOI:** 10.1107/S160053680905377X

**Published:** 2009-12-19

**Authors:** Feng Zhang, Fei-Long Hu, Zhong-Jing Huang, Ji-Chang Zhuang, Yue Zhuang

**Affiliations:** a Department of Chemistry, Guangxi University for Nationalities, Nanning 530006, People’s Republic of China

## Abstract

In the title complex, [Ni_2_(C_13_H_11_F_2_N_6_O)_2_(C_2_H_3_O_2_)_2_]·0.5CH_3_OH, there are two half-molecules in the asymmetric unit. The two centrosymmetrically related Ni^II^ atoms, each attached to an acetate ligand, are linked by two fluconazole ligands. Each Ni^II^ atom is six-coordinated in a distorted octa­hedral geometry by two N atoms of the triazole groups and two bridging O atoms from two different fluconazole ligands and two O atoms from a chelating acetate ligand. In the crystal structure, the half-occupied methanol solvent mol­ecule is linked to a triazole group *via* an O—H⋯N hydrogen bond.

## Related literature

Fluconazole, 2-(2,4-difluoro­phen­yl)-1,3-bis­(1,2,4-triazol-1-yl)-propan-2-ol, is used to treat invasive infections and is an effective agent in preventing invasive infections in patients undergoing bone marrow transplantation, see: Goodman *et al.* (1992 ▶). For general background to inter­actions between metal ions and drugs, see: Agh-Atabay *et al.* (2003 ▶); Ali *et al.* (2002 ▶); Castilo-Blum & Barba-Behrens (2000 ▶); Inoue *et al.* (2002 ▶); Patel *et al.* (2002 ▶); Tavman *et al.* (2000 ▶).
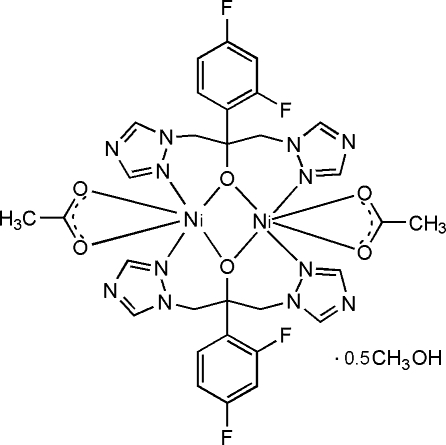


## Experimental

### 

#### Crystal data

[Ni_2_(C_13_H_11_F_2_N_6_O)_2_(C_2_H_3_O_2_)_2_]·0.5CH_4_O
*M* *_r_* = 862.08
Triclinic, 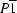

*a* = 11.3898 (12) Å
*b* = 12.4447 (14) Å
*c* = 14.0012 (16) Å
α = 65.211 (1)°
β = 86.815 (2)°
γ = 88.100 (2)°
*V* = 1798.8 (3) Å^3^
*Z* = 2
Mo *K*α radiation
μ = 1.13 mm^−1^
*T* = 298 K
0.42 × 0.37 × 0.35 mm

#### Data collection

Siemens SMART 1000 CCD diffractometer
Absorption correction: multi-scan (*SADABS*; Sheldrick, 1996 ▶) *T*_min_ = 0.648, *T*_max_ = 0.693
9439 measured reflections
6256 independent reflections
4649 reflections with *I* > 2σ(*I*)
*R*_int_ = 0.018

#### Refinement

*R*[*F* ^2^ > 2σ(*F* ^2^)] = 0.043
*wR*(*F* ^2^) = 0.127
*S* = 0.97
6256 reflections
508 parameters
H-atom parameters constrained
Δρ_max_ = 1.09 e Å^−3^
Δρ_min_ = −0.30 e Å^−3^

### 

Data collection: *SMART* (Siemens, 1996 ▶); cell refinement: *SAINT* (Siemens, 1996 ▶); data reduction: *SAINT*; program(s) used to solve structure: *SHELXS97* (Sheldrick, 2008 ▶); program(s) used to refine structure: *SHELXL97* (Sheldrick, 2008 ▶); molecular graphics: *SHELXTL* (Sheldrick, 2008 ▶); software used to prepare material for publication: *SHELXTL*.

## Supplementary Material

Crystal structure: contains datablocks I, global. DOI: 10.1107/S160053680905377X/hy2245sup1.cif

Structure factors: contains datablocks I. DOI: 10.1107/S160053680905377X/hy2245Isup2.hkl

Additional supplementary materials:  crystallographic information; 3D view; checkCIF report

## Figures and Tables

**Table 1:** Selected bond lengths (Å)

Ni1—O1	2.008 (3)
Ni1—O1^i^	2.053 (3)
Ni1—O3	2.050 (3)
Ni1—O4	2.202 (3)
Ni1—N2	2.085 (3)
Ni1—N5^i^	2.076 (3)
Ni2—O2	2.033 (3)
Ni2—O2^ii^	2.030 (3)
Ni2—O5	2.112 (3)
Ni2—O6	2.108 (3)
Ni2—N8	2.089 (3)
Ni2—N11^ii^	2.067 (3)

Symmetry codes: (i) 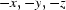; (ii) 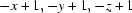.

**Table 2:** Hydrogen-bond geometry (Å, °)

*D*—H⋯*A*	*D*—H	H⋯*A*	*D*⋯*A*	*D*—H⋯*A*
O7—H7⋯N9^iii^	0.82	2.06	2.861 (11)	166

Symmetry code: (iii) 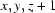.

**Fig. 1.**
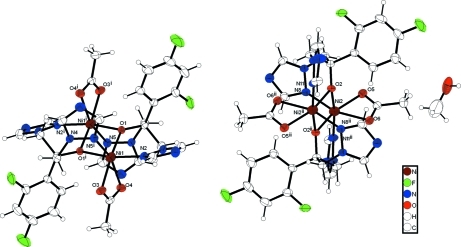
Molecular structure of the title compound. [Symmetry codes: (i) -x, -y, -z; (ii) 1-x, 1-y, 1-z.]

**Fig. 2.**
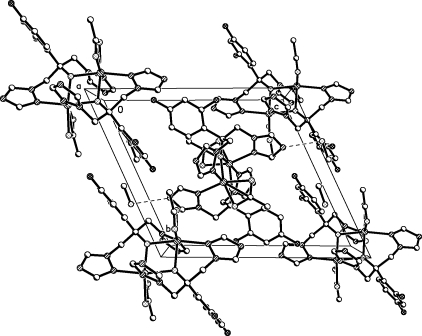
Crystal packing of the title compound, viewed down the a axis. Dashed lines indicate hydrongen bonds. H atoms have been omitted.

## supplementary crystallographic information

### Comment

Fluconazole, 2-(2,4-difluorophenyl)-1,3-bis(1,2,4-triazol-1-yl)-propan-2-ol, is one of the first-line popular drugs used to treat invasive infections and is an effective way in preventing invasive infections in patients undergoing bone marrow transplantation (Goodman *et al.*,  1992). With two symmetrical 1,2,4-triazole groups, fluconazole shows structural flexibility and can form stable complexes with various transition metal ions. Recent years, interactions between metal ions and drugs have become of great interesting (Ali *et al.*, 2002). In some ways, the highest activity of a drug is associated with the existence of a metal ion (Agh-Atabay *et al.*, 2003; Castilo-Blum & Barba-Behrens, 2000; Inoue *et al.*,  2002; Patel *et al.*,  2002; Tavman *et al.*,  2000). We report here the structure of the title compound.

The molecular structure of the complex is shown in Fig. 1. The asymmetric unit contains two Ni^II^ ions, two fluconazole ligands, two bidentate coordinated acetate ligands and a half-occupied solvent methanol molecule. Each fluconazole ligand links the Ni^II^ centers via its deprotonated hydroxyl group and two triazole groups. The Ni^II^ center exhibits a distorted octahedral geometry, defined by two N atoms of the triazole ligands and two O atoms of the deprotonated hydroxyl groups from two different fluconazole ligands, and two O atoms from the acetate ligand (Table 1). The Ni1···Ni1^i^ and Ni2···Ni2^ii^ distances are 3.0974 (7) and 3.0735 (7) Å, respectively [symmetry codes: (i) -x, -y, -z; (ii) 1-x, 1-y, 1-z]. The dihedral angles between the two triazole planes in the same fluconazole ligand are 65.6 (2) and 64.5 (2)°. The two opposite triazole planes in different ligands are parallel. As shown in Fig. 2 and Table 2, hydrogen bond is observed between the triazole group and the methanol molecule.

### Experimental

A mixture of Ni(CH_3_CO_2_)_2_.4H_2_O (0.125 g, 0.5 mmol), fluconazole (0.153 g, 0.5 mmol) and methanol (15 ml) was heated in a Teflon-lined steel bomb at 423K for 3 d. The green crystals were collected, washed with DMF and dried in air. Analysis, calculated for C_30.5_H_30_F_4_N_12_Ni_2_O_6.5_: C 42.48, N 19.50, H 3.48%; found: C 42.61, N 19.41, H 3.53%.

### Refinement

H atoms were positioned geometrically and refined as riding atoms, with C—H = 0.93 (aromatic), 0.97 (CH_2_) and 0.96 (CH_3_) Å and with *U*_iso_(H) = 1.2(1.5 for methyl)*U*_eq_(C). H atom bonded to O atom was found in difference Fourier map and refined as riding, with O—H = 0.82 Å and *U*_iso_(H) = 1.5*U*_eq_(O). The highest residual electron density was found 1.09 Å from O7 and the deepest hole 0.30 Å from C31.

### Crystal data

**Table:**

[Ni_2_(C_13_H_11_F_2_N_6_O)_2_(C_2_H_3_O_2_)_2_]·0.5CH_4_O	*Z* = 2
*M**_r_* = 862.08	*F*(000) = 882
Triclinic, *P*1	*D*_x_ = 1.592 Mg m^−^^3^
Hall symbol: -P 1	Mo *K*α radiation, λ = 0.71073 Å
*a* = 11.3898 (12) Å	Cell parameters from 3550 reflections
*b* = 12.4447 (14) Å	θ = 2.5–27.9°
*c* = 14.0012 (16) Å	µ = 1.13 mm^−^^1^
α = 65.211 (1)°	*T* = 298 K
β = 86.815 (2)°	Block, green
γ = 88.100 (2)°	0.42 × 0.37 × 0.35 mm
*V* = 1798.8 (3) Å^3^	

### Data collection

**Table:**

Siemens SMART 1000 CCD diffractometer	6256 independent reflections
Radiation source: fine-focus sealed tube	4649 reflections with *I* > 2σ(*I*)
graphite	*R*_int_ = 0.018
φ and ω scans	θ_max_ = 25.0°, θ_min_ = 1.6°
Absorption correction: multi-scan (*SADABS*; Sheldrick, 1996)	*h* = −13→11
*T*_min_ = 0.648, *T*_max_ = 0.693	*k* = −13→14
9439 measured reflections	*l* = −16→16

### Refinement

**Table:**

Refinement on *F*^2^	Primary atom site location: structure-invariant direct methods
Least-squares matrix: full	Secondary atom site location: difference Fourier map
*R*[*F*^2^ > 2σ(*F*^2^)] = 0.043	Hydrogen site location: inferred from neighbouring sites
*wR*(*F*^2^) = 0.127	H-atom parameters constrained
*S* = 0.97	*w* = 1/[σ^2^(*F*_o_^2^) + (0.058*P*)^2^ + 3.8657*P*] where *P* = (*F*_o_^2^ + 2*F*_c_^2^)/3
6256 reflections	(Δ/σ)_max_ = 0.001
508 parameters	Δρ_max_ = 1.09 e Å^−^^3^
0 restraints	Δρ_min_ = −0.30 e Å^−^^3^

### Fractional atomic coordinates and isotropic or equivalent isotropic displacement parameters (Å^2^)

**Table:**

	*x*	*y*	*z*	*U*_iso_*/*U*_eq_	Occ. (<1)
Ni1	−0.01760 (4)	−0.09636 (4)	0.11391 (4)	0.03078 (15)	
Ni2	0.62054 (4)	0.55904 (4)	0.46306 (4)	0.03113 (15)	
F1	0.1595 (3)	0.3241 (3)	0.1388 (3)	0.0802 (10)	
F2	−0.2181 (4)	0.4782 (4)	0.1417 (4)	0.1098 (14)	
F3	0.3015 (3)	0.9504 (3)	0.3828 (2)	0.0719 (9)	
F4	0.3922 (4)	0.9592 (3)	0.7008 (3)	0.1012 (13)	
N1	0.0516 (3)	0.0015 (3)	0.2632 (3)	0.0373 (8)	
N2	−0.0209 (3)	−0.0787 (3)	0.2557 (3)	0.0377 (8)	
N3	−0.0622 (4)	−0.0612 (4)	0.4067 (3)	0.0528 (10)	
N7	0.4966 (3)	0.7223 (3)	0.2737 (3)	0.0348 (8)	
N8	0.5773 (3)	0.6318 (3)	0.3051 (3)	0.0372 (8)	
N9	0.5101 (4)	0.6616 (4)	0.1477 (3)	0.0507 (10)	
O1	0.0197 (2)	0.0766 (2)	0.03910 (19)	0.0294 (6)	
O2	0.4526 (2)	0.6025 (2)	0.4913 (2)	0.0311 (6)	
O3	−0.0020 (3)	−0.2773 (3)	0.1774 (2)	0.0446 (7)	
O4	0.1604 (3)	−0.1751 (2)	0.1378 (2)	0.0421 (7)	
O5	0.7026 (3)	0.7080 (3)	0.4645 (2)	0.0421 (7)	
O6	0.6725 (3)	0.5479 (2)	0.6091 (2)	0.0401 (7)	
O7	0.5255 (11)	0.7012 (13)	0.9308 (7)	0.121 (4)	0.50
H7	0.5119	0.6988	0.9897	0.181*	0.50
C1	−0.0878 (4)	−0.1130 (4)	0.3437 (3)	0.0454 (11)	
H1	−0.1473	−0.1682	0.3603	0.054*	
C2	0.0249 (4)	0.0102 (4)	0.3538 (3)	0.0470 (11)	
H2	0.0625	0.0598	0.3766	0.056*	
C3	0.1393 (4)	0.0623 (4)	0.1802 (3)	0.0373 (10)	
H3A	0.1898	0.0041	0.1686	0.045*	
H3B	0.1879	0.1094	0.2025	0.045*	
C4	0.0834 (3)	0.1436 (3)	0.0756 (3)	0.0319 (9)	
C8	0.0046 (4)	0.2366 (4)	0.0923 (3)	0.0373 (10)	
C9	0.0427 (4)	0.3203 (4)	0.1237 (4)	0.0502 (12)	
C10	−0.0297 (5)	0.4014 (5)	0.1419 (5)	0.0709 (17)	
H10	−0.0005	0.4559	0.1637	0.085*	
C11	−0.1454 (5)	0.3975 (5)	0.1262 (5)	0.0693 (16)	
C12	−0.1914 (5)	0.3185 (5)	0.0951 (5)	0.0607 (14)	
H12	−0.2714	0.3182	0.0852	0.073*	
C13	−0.1144 (4)	0.2385 (4)	0.0785 (4)	0.0444 (11)	
H13	−0.1444	0.1839	0.0572	0.053*	
C14	0.1097 (4)	−0.2731 (4)	0.1687 (3)	0.0409 (10)	
C15	0.1762 (5)	−0.3857 (4)	0.1921 (4)	0.0600 (14)	
H15A	0.1644	−0.4123	0.1382	0.090*	
H15B	0.1485	−0.4447	0.2591	0.090*	
H15C	0.2584	−0.3727	0.1943	0.090*	
C16	0.5812 (4)	0.5993 (4)	0.2270 (3)	0.0443 (11)	
H16	0.6293	0.5377	0.2264	0.053*	
C17	0.4589 (4)	0.7378 (4)	0.1811 (3)	0.0426 (10)	
H17	0.4037	0.7948	0.1441	0.051*	
C18	0.4701 (4)	0.7880 (3)	0.3375 (3)	0.0353 (9)	
H18A	0.4257	0.8591	0.2970	0.042*	
H18B	0.5430	0.8118	0.3553	0.042*	
C19	0.3983 (3)	0.7124 (3)	0.4401 (3)	0.0325 (9)	
C23	0.3932 (4)	0.7800 (4)	0.5102 (3)	0.0370 (10)	
C24	0.3472 (4)	0.8938 (4)	0.4802 (4)	0.0489 (12)	
C25	0.3445 (5)	0.9556 (5)	0.5418 (4)	0.0638 (15)	
H25	0.3127	1.0317	0.5188	0.077*	
C26	0.3907 (5)	0.8997 (5)	0.6383 (5)	0.0643 (15)	
C27	0.4371 (5)	0.7879 (5)	0.6747 (4)	0.0603 (14)	
H27	0.4677	0.7520	0.7412	0.072*	
C28	0.4374 (4)	0.7296 (4)	0.6095 (3)	0.0461 (11)	
H28	0.4686	0.6533	0.6337	0.055*	
C29	0.7118 (4)	0.6521 (4)	0.5628 (4)	0.0407 (10)	
C30	0.7676 (5)	0.7072 (5)	0.6254 (4)	0.0596 (14)	
H30A	0.8297	0.6565	0.6646	0.089*	
H30B	0.7096	0.7180	0.6732	0.089*	
H30C	0.7994	0.7827	0.5786	0.089*	
C31	0.554 (2)	0.585 (2)	0.9393 (17)	0.160 (9)	0.50
H31A	0.6279	0.5598	0.9716	0.240*	0.50
H31B	0.5588	0.5856	0.8703	0.240*	0.50
H31C	0.4936	0.5312	0.9816	0.240*	0.50
N10	0.1984 (3)	0.6289 (3)	0.5011 (3)	0.0421 (9)	
N11	0.2213 (3)	0.5129 (3)	0.5628 (3)	0.0390 (8)	
N12	0.0551 (4)	0.5703 (4)	0.6210 (4)	0.0704 (14)	
C20	0.2747 (4)	0.6961 (4)	0.4094 (3)	0.0409 (10)	
H20A	0.2394	0.7732	0.3703	0.049*	
H20B	0.2814	0.6552	0.3636	0.049*	
C21	0.1330 (4)	0.4827 (4)	0.6329 (4)	0.0519 (12)	
H21	0.1250	0.4069	0.6864	0.062*	
H22	0.0654	0.7341	0.5096	0.062*	
C22	0.0994 (5)	0.6589 (5)	0.5386 (4)	0.0637 (15)	
N4	0.2486 (3)	0.1225 (3)	−0.0388 (3)	0.0332 (8)	
N5	0.1993 (3)	0.0854 (3)	−0.1060 (3)	0.0377 (8)	
N6	0.3799 (3)	0.0060 (4)	−0.0680 (3)	0.0539 (10)	
C5	0.1863 (4)	0.2054 (4)	−0.0048 (3)	0.0359 (9)	
H5A	0.1556	0.2707	−0.0655	0.043*	
H5B	0.2406	0.2373	0.0275	0.043*	
C6	0.2825 (4)	0.0161 (4)	−0.1201 (4)	0.0467 (11)	
H6	0.2737	−0.0229	−0.1632	0.056*	
C7	0.3550 (4)	0.0744 (4)	−0.0177 (4)	0.0461 (11)	
H7A	0.4056	0.0868	0.0265	0.055*	

### Atomic displacement parameters (Å^2^)

**Table:**

	*U*^11^	*U*^22^	*U*^33^	*U*^12^	*U*^13^	*U*^23^
Ni1	0.0273 (3)	0.0317 (3)	0.0295 (3)	−0.0020 (2)	−0.0011 (2)	−0.0089 (2)
Ni2	0.0284 (3)	0.0302 (3)	0.0311 (3)	0.0001 (2)	0.0014 (2)	−0.0095 (2)
F1	0.056 (2)	0.104 (3)	0.123 (3)	−0.0002 (17)	−0.0196 (19)	−0.088 (2)
F2	0.095 (3)	0.112 (3)	0.165 (4)	0.031 (2)	0.008 (3)	−0.103 (3)
F3	0.094 (2)	0.0541 (18)	0.066 (2)	0.0376 (17)	−0.0221 (17)	−0.0239 (16)
F4	0.161 (4)	0.088 (3)	0.084 (3)	0.006 (2)	0.010 (2)	−0.067 (2)
N1	0.0351 (19)	0.042 (2)	0.0330 (19)	−0.0058 (16)	−0.0044 (15)	−0.0137 (16)
N2	0.036 (2)	0.043 (2)	0.0312 (18)	−0.0036 (16)	−0.0030 (15)	−0.0123 (16)
N3	0.056 (3)	0.063 (3)	0.039 (2)	−0.008 (2)	0.0062 (19)	−0.022 (2)
N7	0.0362 (19)	0.0324 (18)	0.0304 (18)	0.0010 (15)	0.0033 (15)	−0.0085 (15)
N8	0.037 (2)	0.038 (2)	0.0330 (19)	0.0040 (16)	0.0025 (15)	−0.0121 (16)
N9	0.059 (3)	0.054 (2)	0.039 (2)	0.004 (2)	−0.0032 (19)	−0.0193 (19)
O1	0.0304 (14)	0.0292 (14)	0.0286 (14)	−0.0036 (11)	−0.0009 (11)	−0.0118 (11)
O2	0.0297 (14)	0.0263 (14)	0.0338 (15)	0.0024 (11)	0.0009 (11)	−0.0094 (12)
O3	0.0375 (18)	0.0379 (17)	0.0458 (18)	−0.0038 (13)	−0.0062 (14)	−0.0044 (14)
O4	0.0372 (17)	0.0329 (16)	0.0531 (18)	−0.0034 (13)	−0.0038 (14)	−0.0146 (14)
O5	0.0420 (17)	0.0402 (17)	0.0379 (17)	−0.0049 (13)	−0.0026 (13)	−0.0099 (14)
O6	0.0422 (17)	0.0387 (17)	0.0360 (16)	0.0000 (13)	−0.0043 (13)	−0.0119 (13)
O7	0.147 (10)	0.180 (12)	0.052 (5)	−0.008 (9)	−0.021 (6)	−0.062 (7)
C1	0.044 (3)	0.052 (3)	0.034 (2)	−0.009 (2)	0.002 (2)	−0.012 (2)
C2	0.051 (3)	0.052 (3)	0.040 (3)	−0.004 (2)	−0.002 (2)	−0.021 (2)
C3	0.029 (2)	0.043 (2)	0.037 (2)	−0.0041 (18)	−0.0032 (17)	−0.0137 (19)
C4	0.029 (2)	0.034 (2)	0.035 (2)	−0.0022 (17)	−0.0012 (17)	−0.0154 (18)
C8	0.036 (2)	0.039 (2)	0.039 (2)	0.0019 (18)	0.0000 (18)	−0.018 (2)
C9	0.046 (3)	0.058 (3)	0.060 (3)	−0.001 (2)	−0.001 (2)	−0.038 (3)
C10	0.074 (4)	0.070 (4)	0.096 (5)	−0.001 (3)	0.005 (3)	−0.062 (4)
C11	0.067 (4)	0.066 (4)	0.089 (4)	0.017 (3)	0.010 (3)	−0.050 (3)
C12	0.045 (3)	0.063 (3)	0.081 (4)	0.010 (2)	0.005 (3)	−0.039 (3)
C13	0.037 (3)	0.045 (3)	0.055 (3)	0.001 (2)	0.002 (2)	−0.026 (2)
C14	0.043 (3)	0.037 (2)	0.038 (2)	0.006 (2)	−0.0127 (19)	−0.010 (2)
C15	0.058 (3)	0.042 (3)	0.078 (4)	0.011 (2)	−0.027 (3)	−0.021 (3)
C16	0.052 (3)	0.042 (3)	0.040 (3)	0.006 (2)	0.005 (2)	−0.019 (2)
C17	0.044 (3)	0.045 (3)	0.032 (2)	0.006 (2)	−0.0033 (19)	−0.010 (2)
C18	0.038 (2)	0.031 (2)	0.033 (2)	0.0030 (18)	0.0007 (18)	−0.0102 (18)
C19	0.032 (2)	0.028 (2)	0.034 (2)	0.0037 (16)	−0.0014 (17)	−0.0104 (17)
C23	0.037 (2)	0.037 (2)	0.036 (2)	0.0045 (18)	0.0030 (18)	−0.0158 (19)
C24	0.057 (3)	0.044 (3)	0.045 (3)	0.012 (2)	−0.002 (2)	−0.018 (2)
C25	0.082 (4)	0.046 (3)	0.066 (4)	0.015 (3)	0.009 (3)	−0.028 (3)
C26	0.083 (4)	0.058 (3)	0.065 (4)	0.000 (3)	0.016 (3)	−0.042 (3)
C27	0.081 (4)	0.059 (3)	0.046 (3)	0.005 (3)	0.001 (3)	−0.027 (3)
C28	0.054 (3)	0.045 (3)	0.040 (3)	0.003 (2)	0.003 (2)	−0.020 (2)
C29	0.034 (2)	0.041 (3)	0.049 (3)	0.0045 (19)	−0.0088 (19)	−0.020 (2)
C30	0.067 (4)	0.056 (3)	0.062 (3)	0.000 (3)	−0.018 (3)	−0.029 (3)
C31	0.17 (2)	0.19 (2)	0.128 (17)	−0.004 (18)	−0.048 (15)	−0.066 (17)
N10	0.033 (2)	0.040 (2)	0.045 (2)	0.0059 (16)	0.0001 (16)	−0.0105 (17)
N11	0.0305 (19)	0.037 (2)	0.041 (2)	0.0028 (15)	0.0041 (15)	−0.0082 (16)
N12	0.049 (3)	0.065 (3)	0.074 (3)	0.013 (2)	0.019 (2)	−0.011 (2)
C20	0.040 (2)	0.036 (2)	0.035 (2)	0.0034 (19)	−0.0007 (19)	−0.0040 (19)
C21	0.039 (3)	0.050 (3)	0.053 (3)	0.001 (2)	0.008 (2)	−0.009 (2)
C22	0.045 (3)	0.059 (3)	0.071 (4)	0.020 (3)	0.006 (3)	−0.013 (3)
N4	0.0221 (17)	0.0383 (19)	0.0385 (19)	−0.0034 (14)	−0.0008 (14)	−0.0153 (16)
N5	0.0255 (18)	0.047 (2)	0.043 (2)	−0.0037 (15)	0.0036 (15)	−0.0224 (17)
N6	0.032 (2)	0.062 (3)	0.072 (3)	0.0084 (18)	0.0005 (19)	−0.033 (2)
C5	0.032 (2)	0.036 (2)	0.040 (2)	−0.0021 (18)	0.0008 (18)	−0.0162 (19)
C6	0.035 (3)	0.054 (3)	0.057 (3)	0.000 (2)	0.008 (2)	−0.031 (2)
C7	0.029 (2)	0.054 (3)	0.054 (3)	−0.004 (2)	−0.001 (2)	−0.021 (2)

### Geometric parameters (Å, °)

**Table:**

Ni1—O1	2.008 (3)	C12—H12	0.9300
Ni1—O1^i^	2.053 (3)	C13—H13	0.9300
Ni1—O3	2.050 (3)	C14—C15	1.489 (6)
Ni1—O4	2.202 (3)	C15—H15A	0.9600
Ni1—N2	2.085 (3)	C15—H15B	0.9600
Ni1—N5^i^	2.076 (3)	C15—H15C	0.9600
Ni2—O2	2.033 (3)	C16—H16	0.9300
Ni2—O2^ii^	2.030 (3)	C17—H17	0.9300
Ni2—O5	2.112 (3)	C18—C19	1.552 (5)
Ni2—O6	2.108 (3)	C18—H18A	0.9700
Ni2—N8	2.089 (3)	C18—H18B	0.9700
Ni2—N11^ii^	2.067 (3)	C19—C23	1.535 (6)
F1—C9	1.364 (6)	C19—C20	1.538 (6)
F2—C11	1.360 (6)	C23—C28	1.381 (6)
F3—C24	1.368 (5)	C23—C24	1.390 (6)
F4—C26	1.365 (6)	C24—C25	1.375 (7)
N1—C2	1.335 (5)	C25—C26	1.360 (8)
N1—N2	1.357 (5)	C25—H25	0.9300
N1—C3	1.450 (5)	C26—C27	1.365 (7)
N2—C1	1.325 (5)	C27—C28	1.383 (6)
N3—C2	1.321 (6)	C27—H27	0.9300
N3—C1	1.340 (6)	C28—H28	0.9300
N7—C17	1.323 (5)	C29—C30	1.497 (6)
N7—N8	1.367 (5)	C30—H30A	0.9600
N7—C18	1.457 (5)	C30—H30B	0.9600
N8—C16	1.314 (5)	C30—H30C	0.9600
N9—C17	1.328 (6)	C31—H31A	0.9600
N9—C16	1.351 (6)	C31—H31B	0.9600
O1—C4	1.387 (5)	C31—H31C	0.9600
O1—Ni1^i^	2.053 (3)	N10—C22	1.325 (6)
O2—C19	1.392 (4)	N10—N11	1.361 (5)
O2—Ni2^ii^	2.030 (3)	N10—C20	1.459 (5)
O3—C14	1.272 (5)	N11—C21	1.312 (5)
O4—C14	1.259 (5)	N11—Ni2^ii^	2.067 (3)
O5—C29	1.264 (5)	N12—C22	1.307 (7)
O6—C29	1.266 (5)	N12—C21	1.342 (6)
O7—C31	1.43 (2)	C20—H20A	0.9700
O7—H7	0.8200	C20—H20B	0.9700
C1—H1	0.9300	C21—H21	0.9300
C2—H2	0.9300	C22—H22	0.9300
C3—C4	1.547 (6)	N4—C7	1.326 (5)
C3—H3A	0.9700	N4—N5	1.363 (5)
C3—H3B	0.9700	N4—C5	1.456 (5)
C4—C8	1.527 (6)	N5—C6	1.322 (5)
C4—C5	1.556 (5)	N5—Ni1^i^	2.076 (3)
C8—C13	1.378 (6)	N6—C7	1.329 (6)
C8—C9	1.380 (6)	N6—C6	1.336 (6)
C9—C10	1.378 (7)	C5—H5A	0.9700
C10—C11	1.354 (8)	C5—H5B	0.9700
C10—H10	0.9300	C6—H6	0.9300
C11—C12	1.360 (8)	C7—H7A	0.9300
C12—C13	1.389 (6)		
			
O1—Ni1—O3	162.20 (11)	C14—C15—H15B	109.5
O1—Ni1—O1^i^	80.59 (11)	H15A—C15—H15B	109.5
O3—Ni1—O1^i^	95.01 (11)	C14—C15—H15C	109.5
O1—Ni1—N5^i^	99.07 (12)	H15A—C15—H15C	109.5
O3—Ni1—N5^i^	97.64 (13)	H15B—C15—H15C	109.5
O1^i^—Ni1—N5^i^	84.42 (12)	N8—C16—N9	115.2 (4)
O1—Ni1—N2	88.33 (12)	N8—C16—H16	122.4
O3—Ni1—N2	96.80 (13)	N9—C16—H16	122.4
O1^i^—Ni1—N2	168.17 (12)	N7—C17—N9	111.2 (4)
N5^i^—Ni1—N2	93.32 (14)	N7—C17—H17	124.4
O1—Ni1—O4	101.03 (10)	N9—C17—H17	124.4
O3—Ni1—O4	61.87 (11)	N7—C18—C19	111.4 (3)
O1^i^—Ni1—O4	93.99 (11)	N7—C18—H18A	109.4
N5^i^—Ni1—O4	159.30 (12)	C19—C18—H18A	109.4
N2—Ni1—O4	92.17 (13)	N7—C18—H18B	109.4
O2^ii^—Ni2—O2	81.69 (11)	C19—C18—H18B	109.4
O2^ii^—Ni2—N11^ii^	88.38 (12)	H18A—C18—H18B	108.0
O2—Ni2—N11^ii^	169.91 (12)	O2—C19—C23	110.3 (3)
O2^ii^—Ni2—N8	98.46 (12)	O2—C19—C20	109.8 (3)
O2—Ni2—N8	85.09 (12)	C23—C19—C20	111.4 (3)
N11^ii^—Ni2—N8	94.62 (14)	O2—C19—C18	110.0 (3)
O2^ii^—Ni2—O6	100.48 (11)	C23—C19—C18	107.5 (3)
O2—Ni2—O6	91.88 (11)	C20—C19—C18	107.8 (3)
N11^ii^—Ni2—O6	91.72 (13)	C28—C23—C24	115.2 (4)
N8—Ni2—O6	160.18 (13)	C28—C23—C19	120.6 (4)
O2^ii^—Ni2—O5	162.83 (11)	C24—C23—C19	124.2 (4)
O2—Ni2—O5	97.31 (11)	F3—C24—C25	116.6 (4)
N11^ii^—Ni2—O5	92.72 (13)	F3—C24—C23	119.0 (4)
N8—Ni2—O5	98.53 (12)	C25—C24—C23	124.4 (5)
O6—Ni2—O5	62.37 (11)	C26—C25—C24	116.5 (5)
C2—N1—N2	108.4 (3)	C26—C25—H25	121.7
C2—N1—C3	130.6 (4)	C24—C25—H25	121.7
N2—N1—C3	120.9 (3)	C25—C26—C27	123.2 (5)
C1—N2—N1	103.3 (3)	C25—C26—F4	118.3 (5)
C1—N2—Ni1	137.8 (3)	C27—C26—F4	118.5 (5)
N1—N2—Ni1	118.1 (2)	C26—C27—C28	117.9 (5)
C2—N3—C1	103.2 (4)	C26—C27—H27	121.1
C17—N7—N8	109.0 (3)	C28—C27—H27	121.1
C17—N7—C18	130.7 (4)	C23—C28—C27	122.8 (5)
N8—N7—C18	120.2 (3)	C23—C28—H28	118.6
C16—N8—N7	102.5 (3)	C27—C28—H28	118.6
C16—N8—Ni2	137.4 (3)	O5—C29—O6	119.4 (4)
N7—N8—Ni2	117.4 (2)	O5—C29—C30	121.1 (4)
C17—N9—C16	102.1 (4)	O6—C29—C30	119.5 (4)
C4—O1—Ni1	127.5 (2)	C29—C30—H30A	109.5
C4—O1—Ni1^i^	126.3 (2)	C29—C30—H30B	109.5
Ni1—O1—Ni1^i^	99.41 (11)	H30A—C30—H30B	109.5
C19—O2—Ni2^ii^	127.2 (2)	C29—C30—H30C	109.5
C19—O2—Ni2	126.1 (2)	H30A—C30—H30C	109.5
Ni2^ii^—O2—Ni2	98.31 (11)	H30B—C30—H30C	109.5
C14—O3—Ni1	92.3 (2)	O7—C31—H31A	109.5
C14—O4—Ni1	85.8 (3)	O7—C31—H31B	109.5
C29—O5—Ni2	89.0 (3)	H31A—C31—H31B	109.5
C29—O6—Ni2	89.2 (2)	O7—C31—H31C	109.5
C31—O7—H7	109.5	H31A—C31—H31C	109.5
N2—C1—N3	114.2 (4)	H31B—C31—H31C	109.5
N2—C1—H1	122.9	C22—N10—N11	107.8 (4)
N3—C1—H1	122.9	C22—N10—C20	131.2 (4)
N3—C2—N1	110.9 (4)	N11—N10—C20	120.9 (3)
N3—C2—H2	124.5	C21—N11—N10	103.2 (3)
N1—C2—H2	124.5	C21—N11—Ni2^ii^	138.1 (3)
N1—C3—C4	112.3 (3)	N10—N11—Ni2^ii^	118.2 (2)
N1—C3—H3A	109.2	C22—N12—C21	102.5 (4)
C4—C3—H3A	109.2	N10—C20—C19	112.2 (3)
N1—C3—H3B	109.2	N10—C20—H20A	109.2
C4—C3—H3B	109.2	C19—C20—H20A	109.2
H3A—C3—H3B	107.9	N10—C20—H20B	109.2
O1—C4—C8	110.7 (3)	C19—C20—H20B	109.2
O1—C4—C3	109.9 (3)	H20A—C20—H20B	107.9
C8—C4—C3	109.8 (3)	N11—C21—N12	114.5 (4)
O1—C4—C5	109.7 (3)	N11—C21—H21	122.7
C8—C4—C5	109.8 (3)	N12—C21—H21	122.7
C3—C4—C5	106.8 (3)	N12—C22—N10	112.0 (4)
C13—C8—C9	115.3 (4)	N12—C22—H22	124.0
C13—C8—C4	119.8 (4)	N10—C22—H22	124.0
C9—C8—C4	124.8 (4)	C7—N4—N5	108.8 (3)
F1—C9—C10	116.9 (4)	C7—N4—C5	130.5 (4)
F1—C9—C8	118.7 (4)	N5—N4—C5	120.7 (3)
C10—C9—C8	124.4 (5)	C6—N5—N4	102.5 (3)
C11—C10—C9	116.5 (5)	C6—N5—Ni1^i^	135.2 (3)
C11—C10—H10	121.7	N4—N5—Ni1^i^	117.6 (2)
C9—C10—H10	121.7	C7—N6—C6	102.4 (4)
C10—C11—C12	123.4 (5)	N4—C5—C4	111.0 (3)
C10—C11—F2	117.5 (5)	N4—C5—H5A	109.4
C12—C11—F2	119.1 (6)	C4—C5—H5A	109.4
C11—C12—C13	117.5 (5)	N4—C5—H5B	109.4
C11—C12—H12	121.2	C4—C5—H5B	109.4
C13—C12—H12	121.2	H5A—C5—H5B	108.0
C8—C13—C12	122.8 (5)	N5—C6—N6	115.2 (4)
C8—C13—H13	118.6	N5—C6—H6	122.4
C12—C13—H13	118.6	N6—C6—H6	122.4
O4—C14—O3	119.8 (4)	N4—C7—N6	111.1 (4)
O4—C14—C15	122.1 (4)	N4—C7—H7A	124.5
O3—C14—C15	118.1 (4)	N6—C7—H7A	124.5
C14—C15—H15A	109.5		
			
C2—N1—N2—C1	0.3 (5)	C3—C4—C8—C9	61.4 (5)
C3—N1—N2—C1	−179.3 (4)	C5—C4—C8—C9	−55.8 (6)
C2—N1—N2—Ni1	171.8 (3)	C13—C8—C9—F1	−179.8 (4)
C3—N1—N2—Ni1	−7.7 (5)	C4—C8—C9—F1	1.8 (7)
O1—Ni1—N2—C1	135.6 (5)	C13—C8—C9—C10	0.6 (8)
O3—Ni1—N2—C1	−61.5 (5)	C4—C8—C9—C10	−177.8 (5)
O1^i^—Ni1—N2—C1	115.2 (6)	F1—C9—C10—C11	179.7 (5)
N5^i^—Ni1—N2—C1	36.6 (5)	C8—C9—C10—C11	−0.7 (9)
O4—Ni1—N2—C1	−123.4 (5)	C9—C10—C11—C12	0.5 (10)
O1—Ni1—N2—N1	−32.0 (3)	C9—C10—C11—F2	−178.8 (5)
O3—Ni1—N2—N1	130.9 (3)	C10—C11—C12—C13	−0.2 (10)
O1^i^—Ni1—N2—N1	−52.5 (7)	F2—C11—C12—C13	179.1 (5)
N5^i^—Ni1—N2—N1	−131.0 (3)	C9—C8—C13—C12	−0.2 (7)
O4—Ni1—N2—N1	68.9 (3)	C4—C8—C13—C12	178.3 (4)
C17—N7—N8—C16	−0.3 (4)	C11—C12—C13—C8	0.0 (8)
C18—N7—N8—C16	−177.1 (3)	Ni1—O4—C14—O3	−5.0 (4)
C17—N7—N8—Ni2	−165.0 (3)	Ni1—O4—C14—C15	172.7 (4)
C18—N7—N8—Ni2	18.3 (4)	Ni1—O3—C14—O4	5.3 (4)
O2^ii^—Ni2—N8—C16	−45.9 (5)	Ni1—O3—C14—C15	−172.4 (4)
O2—Ni2—N8—C16	−126.7 (5)	N7—N8—C16—N9	0.3 (5)
N11^ii^—Ni2—N8—C16	43.2 (5)	Ni2—N8—C16—N9	160.0 (3)
O6—Ni2—N8—C16	151.4 (4)	C17—N9—C16—N8	−0.2 (5)
O5—Ni2—N8—C16	136.6 (4)	N8—N7—C17—N9	0.2 (5)
O2^ii^—Ni2—N8—N7	111.7 (3)	C18—N7—C17—N9	176.5 (4)
O2—Ni2—N8—N7	30.9 (3)	C16—N9—C17—N7	0.0 (5)
N11^ii^—Ni2—N8—N7	−159.3 (3)	C17—N7—C18—C19	112.1 (5)
O6—Ni2—N8—N7	−51.0 (5)	N8—N7—C18—C19	−71.9 (4)
O5—Ni2—N8—N7	−65.8 (3)	Ni2^ii^—O2—C19—C23	116.3 (3)
O3—Ni1—O1—C4	−75.3 (5)	Ni2—O2—C19—C23	−102.6 (3)
O1^i^—Ni1—O1—C4	−152.2 (3)	Ni2^ii^—O2—C19—C20	−6.9 (4)
N5^i^—Ni1—O1—C4	125.0 (3)	Ni2—O2—C19—C20	134.2 (3)
N2—Ni1—O1—C4	31.9 (3)	Ni2^ii^—O2—C19—C18	−125.3 (3)
O4—Ni1—O1—C4	−60.0 (3)	Ni2—O2—C19—C18	15.7 (4)
O3—Ni1—O1—Ni1^i^	76.9 (4)	N7—C18—C19—O2	50.3 (4)
O1^i^—Ni1—O1—Ni1^i^	0.0	N7—C18—C19—C23	170.4 (3)
N5^i^—Ni1—O1—Ni1^i^	−82.74 (13)	N7—C18—C19—C20	−69.4 (4)
N2—Ni1—O1—Ni1^i^	−175.84 (13)	O2—C19—C23—C28	−0.9 (5)
O4—Ni1—O1—Ni1^i^	92.27 (12)	C20—C19—C23—C28	121.3 (4)
O2^ii^—Ni2—O2—C19	−149.6 (3)	C18—C19—C23—C28	−120.8 (4)
N11^ii^—Ni2—O2—C19	−139.0 (7)	O2—C19—C23—C24	178.1 (4)
N8—Ni2—O2—C19	−50.3 (3)	C20—C19—C23—C24	−59.6 (5)
O6—Ni2—O2—C19	110.1 (3)	C18—C19—C23—C24	58.2 (5)
O5—Ni2—O2—C19	47.7 (3)	C28—C23—C24—F3	−180.0 (4)
O2^ii^—Ni2—O2—Ni2^ii^	0.0	C19—C23—C24—F3	0.9 (7)
N11^ii^—Ni2—O2—Ni2^ii^	10.5 (7)	C28—C23—C24—C25	0.4 (7)
N8—Ni2—O2—Ni2^ii^	99.31 (13)	C19—C23—C24—C25	−178.7 (5)
O6—Ni2—O2—Ni2^ii^	−100.31 (12)	F3—C24—C25—C26	−179.5 (5)
O5—Ni2—O2—Ni2^ii^	−162.69 (11)	C23—C24—C25—C26	0.1 (8)
O1—Ni1—O3—C14	14.2 (5)	C24—C25—C26—C27	−0.5 (9)
O1^i^—Ni1—O3—C14	88.9 (3)	C24—C25—C26—F4	178.4 (5)
N5^i^—Ni1—O3—C14	173.9 (3)	C25—C26—C27—C28	0.3 (9)
N2—Ni1—O3—C14	−91.8 (3)	F4—C26—C27—C28	−178.6 (5)
O4—Ni1—O3—C14	−3.0 (2)	C24—C23—C28—C27	−0.5 (7)
O1—Ni1—O4—C14	−171.7 (2)	C19—C23—C28—C27	178.6 (4)
O3—Ni1—O4—C14	3.0 (2)	C26—C27—C28—C23	0.2 (8)
O1^i^—Ni1—O4—C14	−90.5 (3)	Ni2—O5—C29—O6	−0.6 (4)
N5^i^—Ni1—O4—C14	−5.7 (5)	Ni2—O5—C29—C30	179.8 (4)
N2—Ni1—O4—C14	99.6 (3)	Ni2—O6—C29—O5	0.6 (4)
O2^ii^—Ni2—O5—C29	3.1 (5)	Ni2—O6—C29—C30	−179.8 (4)
O2—Ni2—O5—C29	88.6 (2)	C22—N10—N11—C21	0.0 (5)
N11^ii^—Ni2—O5—C29	−90.2 (3)	C20—N10—N11—C21	−177.5 (4)
N8—Ni2—O5—C29	174.7 (3)	C22—N10—N11—Ni2^ii^	−173.4 (4)
O6—Ni2—O5—C29	0.3 (2)	C20—N10—N11—Ni2^ii^	9.0 (5)
O2^ii^—Ni2—O6—C29	−179.5 (2)	C22—N10—C20—C19	116.9 (6)
O2—Ni2—O6—C29	−97.6 (2)	N11—N10—C20—C19	−66.2 (5)
N11^ii^—Ni2—O6—C29	91.8 (3)	O2—C19—C20—N10	62.0 (4)
N8—Ni2—O6—C29	−16.9 (5)	C23—C19—C20—N10	−60.5 (4)
O5—Ni2—O6—C29	−0.3 (2)	C18—C19—C20—N10	−178.2 (3)
N1—N2—C1—N3	−0.6 (5)	N10—N11—C21—N12	0.1 (6)
Ni1—N2—C1—N3	−169.4 (3)	Ni2^ii^—N11—C21—N12	171.4 (4)
C2—N3—C1—N2	0.7 (6)	C22—N12—C21—N11	−0.1 (7)
C1—N3—C2—N1	−0.5 (5)	C21—N12—C22—N10	0.1 (7)
N2—N1—C2—N3	0.1 (5)	N11—N10—C22—N12	0.0 (7)
C3—N1—C2—N3	179.6 (4)	C20—N10—C22—N12	177.1 (5)
C2—N1—C3—C4	−114.6 (5)	C7—N4—N5—C6	0.1 (4)
N2—N1—C3—C4	64.8 (5)	C5—N4—N5—C6	178.0 (4)
Ni1—O1—C4—C8	−113.7 (3)	C7—N4—N5—Ni1^i^	159.5 (3)
Ni1^i^—O1—C4—C8	101.1 (3)	C5—N4—N5—Ni1^i^	−22.6 (4)
Ni1—O1—C4—C3	7.8 (4)	C7—N4—C5—C4	−109.0 (5)
Ni1^i^—O1—C4—C3	−137.4 (3)	N5—N4—C5—C4	73.5 (4)
Ni1—O1—C4—C5	125.0 (3)	O1—C4—C5—N4	−46.4 (4)
Ni1^i^—O1—C4—C5	−20.2 (4)	C8—C4—C5—N4	−168.3 (3)
N1—C3—C4—O1	−62.6 (4)	C3—C4—C5—N4	72.7 (4)
N1—C3—C4—C8	59.4 (4)	N4—N5—C6—N6	−0.1 (5)
N1—C3—C4—C5	178.4 (3)	Ni1^i^—N5—C6—N6	−153.8 (3)
O1—C4—C8—C13	4.7 (5)	C7—N6—C6—N5	0.1 (6)
C3—C4—C8—C13	−116.9 (4)	N5—N4—C7—N6	0.0 (5)
C5—C4—C8—C13	125.9 (4)	C5—N4—C7—N6	−177.7 (4)
O1—C4—C8—C9	−177.0 (4)	C6—N6—C7—N4	0.0 (5)

Symmetry codes: (i) −*x*, −*y*, −*z*; (ii) −*x*+1, −*y*+1, −*z*+1.

### Hydrogen-bond geometry (Å, °)

**Table:**

*D*—H···*A*	*D*—H	H···*A*	*D*···*A*	*D*—H···*A*
O7—H7···N9^iii^	0.82	2.06	2.861 (11)	166

Symmetry codes: (iii) *x*, *y*, *z*+1.
